# Hormonal Content and Gene Expression during Olive Fruit Growth and Ripening

**DOI:** 10.3390/plants12223832

**Published:** 2023-11-12

**Authors:** Maria C. Camarero, Beatriz Briegas, Jorge Corbacho, Juana Labrador, Maria C. Gomez-Jimenez

**Affiliations:** Laboratory of Plant Physiology, Universidad de Extremadura, Avda de Elvas s/n, 06006 Badajoz, Spain

**Keywords:** fruit development, fruit ripening, fruit size, gene expression, hormone, olive

## Abstract

The cultivated olive (*Olea europaea* L. subsp. *europaea* var. *europaea*) is one of the most valuable fruit trees worldwide. However, the hormonal mechanisms underlying the fruit growth and ripening in olives remain largely uncharacterized. In this study, we investigated the physiological and hormonal changes, by ultra-high performance liquid chromatography-mass spectrometry (UHPLC-MS), as well as the expression patterns of hormone-related genes, using quantitative real-time PCR (qRT-PCR) analysis, during fruit growth and ripening in two olive cultivars, ‘Arbequina’ and ‘Picual’, with contrasting fruit size and shape as well as fruit ripening duration. Hormonal profiling revealed that olive fruit growth involves a lowering of auxin (IAA), cytokinin (CKs), and jasmonic acid (JA) levels as well as a rise in salicylic acid (SA) levels from the endocarp lignification to the onset of fruit ripening in both cultivars. During olive fruit ripening, both abscisic acid (ABA) and anthocyanin levels rose, while JA levels fell, and SA levels showed no significant changes in either cultivar. By contrast, differential accumulation patterns of gibberellins (GAs) were found between the two cultivars during olive fruit growth and ripening. GA_1_ was not detected at either stage of fruit development in ‘Arbequina’, revealing a specific association between the GA_1_ and ‘Picual’, the cultivar with large sized, elongated, and fast-ripening fruit. Moreover, ABA may play a central role in regulating olive fruit ripening through transcriptional regulation of key ABA metabolism genes, whereas the IAA, CK, and GA levels and/or responsiveness differ between olive cultivars during olive fruit ripening. Taken together, the results indicate that the relative absence or presence of endogenous GA_1_ is associated with differences in fruit morphology and size as well as in the ripening duration in olives. Such detailed knowledge may be of help to design new strategies for effective manipulation of olive fruit size as well as ripening duration.

## 1. Introduction

The cultivated olive (*Olea europaea* L. subsp. *europaea* var. *europaea*), used for the eating the fruit and extracting oil, displays significant phenotypic variation in fruit quality and yield traits [1,2,3,4,5,6,7,8,9,10,11,12,13]. Despite the importance of understanding their control of olive fruit size and ripening, the hormonal regulatory mechanisms underlying olive fruit growth and ripening, as well as the relative roles of the plant hormones on different olive fruit traits, remain poorly understood. Olive fruits are similar to stone fruits in that they share common events in their life cycle, including fertilization and fruit set, early fruit growth, endocarp lignification, late fruit growth, fruit maturation, and ripening [14,15]. This period can oscillate between 5 and 9 months, from anthesis to the fully ripe fruit stage, depending on olive cultivar, climatic conditions, and agricultural practices [1,7,10,14,15,16,17]. The successful fertilization of the ovule is followed by cell division and expansion, resulting in the rapid growth of the olive fruit [18,19,20]. Subsequently, early fruit growth is due solely to cell expansion until endocarp lignification about 50 days following ovary fertilization [20]. Next, the late growth of olive fruit comprises mainly pulp cell expansion, which is responsible for the maximum fruit size, and oil accumulation [14,15]. However, recently, it has been demonstrated that cell division is induced by water deficits in olive fruit during this late fruit growth phase [21]. Finally, the olive fruit undergoes a complex and highly coordinated series of events in the fruit ripening process, which is characterized by gradual increases in oil and anthocyanin contents [14,15,22]. Moreover, the olive fruit ripening process depends on the olive cultivars in terms of different dates of onset of fruit ripening, as well as different ripening durations and rates [1,3,7,9,10,23,24,25,26].

Generally, olive fruits are classified as non-climacteric fruits [27] because previous studies have found no burst of ethylene during olive fruit ripening, and ethylene treatment after harvest showed no olive fruit softening or anthocyanin synthesis [28,29]. However, while information regarding ethylene metabolism in olive fruits is limited, several studies have tracked fruit respiration during olive fruit ripening [30,31,32,33,34], in some cases, reporting contradictory results. Indeed, ethylene is effective in olive fruits, given that the ethylene inhibitors, 1-methyl cyclopropropene (1-MCP) or 1-amino ethoxyvinyl glycine (AVG), can reverse the ethylene responses, but the impact of these treatments or of exogenous ethylene reportedly depend on the olive cultivar [34,35,36,37,38,39]. Moreover, the level of ethylene precursor, 1-aminocyclopropane-1-carboxylic acid (ACC), rises as olive fruit ripens [25], thus suggesting differential fruit ACC levels and ethylene sensitivity between olive cultivars.

Because changes in plant hormone metabolism during fleshy fruit development and ripening may be part of a mechanism controlling the transition from pericarp cell expansion to ripening, hormone levels should be maintained within strict limits [40,41,42,43,44,45,46,47]. Moreover, several studies have indicated that the regulation of fleshy fruit development in both perennial and annual crops, and therefore, the final quality of fleshy fruit involves differential plant hormone levels between cultivars within a single plant species [48,49,50,51,52]. In olives, although the temporal transcriptional, proteomic, and metabolic changes during fruit ripening in different olive cultivars have been well reported [22,53,54,55,56,57,58,59,60,61,62,63,64,65,66,67,68,69], the accumulation dynamics of the different plant hormones during fruit ripening have not previously been described. In particular, high levels of cytokinins (CKs), gibberellins (GAs), and polyamines (PAs) have been reported in the developing olive fruits [5,70,71], whereas abscisic acid (ABA), salicylic acid (SA), and GAs are abundant in the ripe olive fruit [65]. In this context, the preharvest application of plant hormones to different olive cultivars has recently been reported to increase fruit size and to boost the content of bioactive components and antioxidants, among other components, and thus to preserve the oxidative stability of olive fruits during storage [72,73,74,75,76]. Recently, it has been demonstrated that CKs and SA correlate positively with cell division during early olive fruit development, while GA, auxin, and ABA have higher relative importance in the period when olive fruit growth depends mainly on cell expansion [20].

The objective of the present study is to help elucidate the temporal changes in hormonal composition and gene expression that occur during olive fruit growth and ripening. For this purpose, comparative physiological, cytological, and hormonal analyses were conducted for olive cultivars ‘Picual’ and ‘Arbequina’, which have contrasting fruit size and shape as well as ripening duration, together with the expression patterns of hormone-related genes during olive fruit growth and ripening. These data provide a starting point to clarify the hormonal differences of olive fruit growth and ripening among olive cultivars. Collectively, our results highlight the possible role of GA in coordinating the fruit size and the progression of fruit ripening in olives, which would have major biological and practical implications.

## 2. Results

### 2.1. Physiological Changes during Olive Fruit Growth and Ripening

**Figure 1:**
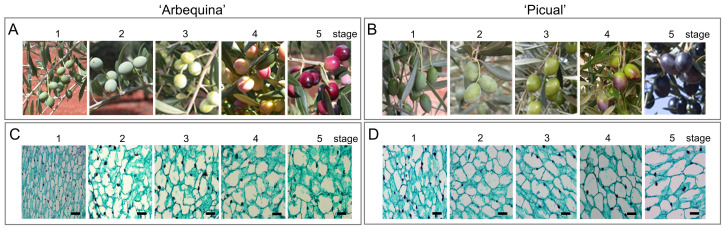
Development of ‘Arbequina’ and ‘Picual’ olive fruits. Morphological changes of olive fruit of cultivars ‘Arbequina’ (**A**) and ‘Picual’ (**B**) during the stages of olive fruit development. Pericarp cross-section of olive fruit of ‘Arbequina’ (**C**) and ‘Picual’ (**D**) during the stages of olive fruit development. Stage 1: ‘the complete pit hardening’; Stage 2: ‘the immature green’ (peak of pericarp cell expansion); Stage 3: ‘the mature green’; Stage 4: ‘the turning’ (the onset of fruit ripening); Stage 5: ‘the fully ripe’. Bars = 100 μm.

**Figure 2:**
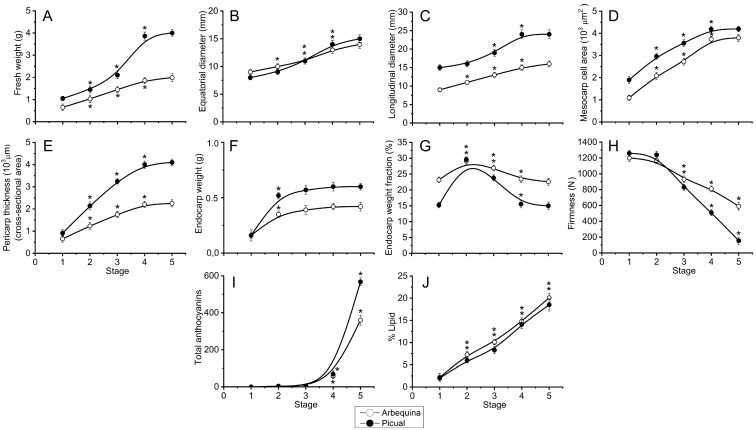
(**A**) Changes in fresh weight (FW) (g fruit^−1^), (**B**) equatorial diameter (mm), (**C**) longitudinal diameter (mm), (**D**) mesocarp cell area, (**E**) pericarp thickness, (**F**) endocarp weight, (**G**) endocarp weight fraction, (**H**) firmness, (**I**) total anthocyanins, and (**J**) % lipid of the ‘Arbequina’ (open circles) and ‘Picual’ (solid circles) fruit during the stages of olive fruit development. Fresh weight, equatorial diameter, longitudinal diameter, and firmness were measured on whole fruits. Anthocyanin and lipid content were measured in fruit pericarps. Fruit shape index is the length-to-width ratio of the fruit. Asterisks indicate statistically significant changes with respect to the preceding point according to Tukey’s test (*p* < 0.05). Data are the means of three independent experiments ± SE.

As observed previously [5], olive cultivars, such as ‘Picual’ and ‘Arbequina’, display significant phenotypic variation in fruit size and shape: ‘Picual’ is an olive cultivar with large sized, elongated fruit, while ‘Arbequina’ bears small sized, round fruit. In the present study, the physiological and cytological changes during fruit development in these two olive cultivars were recorded (Figure 1 and Figure 2). Five major stages were established to define olive fruit development: (1) ‘small immature fruit’ with complete pit hardening, (2) ‘large immature green fruit’, (3) ‘mature green fruit’, (4) ‘turning fruit’, and (5) ‘fully ripe fruit’ (Figure 1A,B). Under the same grown conditions, mature green ‘Picual’ olive fruits required about 50 days for complete ripening, as indicated by the fully black colour and softening of the fruit, whereas mature green ‘Arbequina’ olive fruits required about 90 days. Thus, a marked variability was found in the duration of the fruit ripening process between the two olive cultivars: fast-ripening cultivar (‘Picual’) and slow-ripening cultivar (‘Arbequina’).

The cytological observations and comparison of growth curves indicated that the stages of ‘Arbequina’ fruit are equivalent to that of ‘Picual’ fruit (Figure 1 and Figure 2). The fruit pericarp cells were tightly arranged in young developing fruit (stage 1) of both olive cultivars, but as the fruit pulp expanded (stages 2–4) and ripened (stage 5) the fruit pericarp cells began to lose their close connections. In the fully ripe fruit (stage 5) of both cultivars, pericarp cells had fewer points of attachment to their neighbours and air spaces developed between cells, indicating that intercellular adhesion was declining (Figure 1C,D).

‘Arbequina’ and ‘Picual’ olive fruits followed a highly reproducible progression of growth, including increases in weight, longitudinal and equatorial diameters, mesocarp cell area, and pericarp thickness from stage 1 to 4, while the values remained constant between stages 4 and 5 in both olive cultivars (Figure 2A–E). During the pulp growth phase, olive fruit growth led to a significant increase in fruit weight in both olive cultivars (Figure 2A). Consequently, the fruit weight augmented in ‘Arbequina’ 3-fold (66% of final fruit weight) and in ‘Picual’ 4-fold (75% of final fruit weight) during this phase (Figure 2A). In the ‘Picual’ cultivar, the fruit weight was significantly greater than the ‘Arbequina’ cultivar. In fact, the weight of fully ripe ‘Picual’ fruit reached double that of fully ripe ‘Arbequina’ fruit. In this stage, ‘Picual’ fruit (the cultivar with large, elongated fruit) measured about 15 mm in equatorial diameter and 24 mm in longitudinal diameter, while ‘Arbequina’ fruit (the cultivar with small, round fruit) reached about 14 mm in equatorial diameter and 16 mm in longitudinal diameter. Moreover, in the ‘Arbequina’ cultivar, fruit length and width followed similar trends, resulting in a rounder shape in this cultivar (Figure 2B,C), while in ‘Picual’ the fruit grew more rapidly in length than in width, resulting in the characteristic elongated shape in this cultivar (Figure 2B,C). During this phase, olive fruits increased their size mainly by cell expansion, and thus the mesocarp cell area reached more than 60% of the final mesocarp cell size in both olive cultivars (Figure 2D). Likewise, the endocarp weight was higher in ‘Picual’ than in ‘Arbequina’, a finding consistent with the greater fruit weight observed in ‘Picual’ during fruit growth. However, the dynamics of the endocarp weight and endocarp weight fraction (%) were found to be similar between fruit stages in both olive cultivars (Figure 2F,G).

In both cultivars, olive fruit firmness decreased after the onset of ripening and became oversoft at stage 5 (fully ripe fruit), but fruit firmness rapidly softened and was much softer in ‘Picual’ than in ‘Arbequina’ during fruit ripening (from stage 4 to 5) (Figure 2H). Thus, the degree of softening differed between the cultivars, resulting in unequal final fruit firmness. Conversely, total anthocyanins were undetectable before stage 3, but rapidly increased during fruit ripening (stages 4–5) in both cultivars and were also higher in the fully ripe fruits of ‘Picual’ than in those of ‘Arbequina’ (Figure 2I). Notwithstanding the observed differences in the fruit ripening between the two olive cultivars, the total lipid contents showed similar increasing trends throughout all stages in both olive cultivars (Figure 2J).

### 2.2. Quantitative Changes of Plant Hormones during Olive Fruit Growth and Ripening

**Figure 3:**
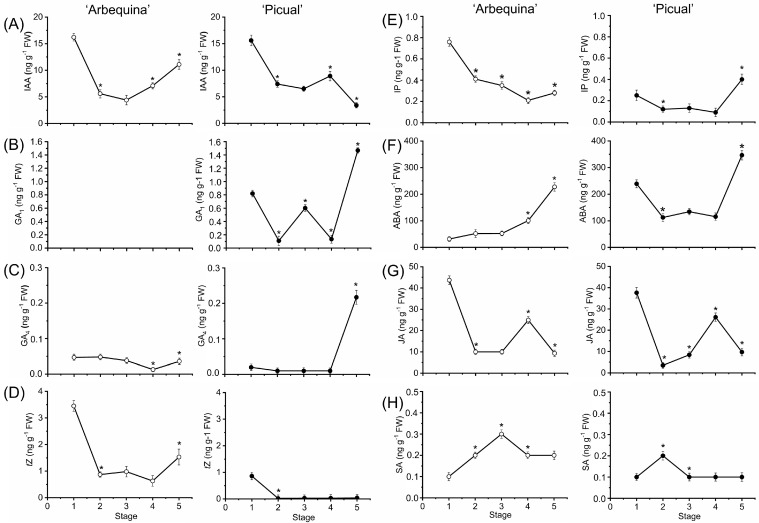
Profiles of IAA (**A**), GA_1_ (**B**), GA_4_ (**C**), *t*Z (**D**), iP (**E**), ABA (**F**), SA (**G**), and JA (**H**) levels [ng g^−1^ fresh weight (FW)] measured from ‘Arbequina’ and ‘Picual’ fruits during the stages of olive fruit development. Data are the means ± SD of three biological replicates with three technical repeats each. Asterisks indicate statistically significant changes with respect to the preceding point according to Tukey’s test (*p* < 0.05). IAA: indole-3-acetic acid; GA: gibberellin; *t*Z: *trans*-Zeatin; iP: isopentenyladenine; ABA: abscisic acid; JA: jasmonic acid; SA: salicylic acid.

In an effort to elucidate the possible relationship between hormone composition and olive fruit development, the hormone profiles of indole-3-acetic acid (IAA), GAs (GA_1_ and GA_4_), CKs [trans-Zeatin (tZ) and isopentenyladenine (iP)], ABA, SA, and jasmonic acid (JA) were analysed in both olive cultivars during fruit growth and ripening. The levels of hormones changed differentially within and between olive fruits of the two cultivars during fruit growth and ripening (Figure 3). By contrast, ethylene levels were not detected in any of the fruit samples (d).

In both olive cultivars, the IAA content of olive fruits was greatest at stage 1 and gradually decreased thereafter, to stage 3 (Figure 3A). In fact, IAA levels fell steadily to as much as 73% and 58% from stage 1 to 3, in ‘Arbequina’ and ‘Picual’ fruit, respectively. From stage 3 to 4, the levels of IAA increased in olive fruit of both cultivars, but the IAA level continued to rise in ‘Arbequina’ fruit from stage 4 to 5, while the IAA level fell by 62% in ‘Picual’ fruit (Figure 3A). Thus, endogenous IAA levels declined during fruit growth, whereas a surge occurred at the onset of ripening in both olive cultivars. However, olive fruit ripening was accompanied by a lower IAA level only in ‘Picual’ fruits (fast-ripening cultivar), which contained lower levels (about 30%) of endogenous IAA than did ‘Arbequina’ fruits (slow-ripening cultivar) at the fully ripe stage.

Notably, in contrast to ‘Picual’ fruit (the cultivar with large, elongated fruit), GA_1_ was not detected at either stage of fruit development in ‘Arbequina’ fruit (the cultivar with small, round fruit) (Figure 3B). In ‘Picual’ fruit, GA_1_ levels fell by 87% from stage 1 to 2, while they rose 6-fold from stage 2 to 3 (Figure 3B). From stage 3 to 4, GA_1_ levels fell up to 78% in ‘Picual’ fruit to rise later on, up to 11-fold, between stage 4 to 5 in ‘Picual’ fruit (Figure 3B). These results identify GA_1_ as the specific bioactive GA of the ‘Picual’ fruit (Figure 3B). On the other hand, in both olive cultivars, endogenous GA_4_ maintained relatively low levels and remained unaltered during fruit growth (stages 1–3), while an increase was detected through stages 4 to 5 only in ‘Picual’ fruit (Figure 3C). Thus, unlike in ‘Arbequina’ fruit, GA_1_ and GA_4_ levels rose in ‘Picual’ fruit at the last stage of fruit ripening (stage 5, fully ripe fruit), when the highest levels were measured (Figure 3B,C), and, consequently, ripe ‘Picual’ fruits (fast-ripening cultivar) were found to contain higher levels of endogenous GAs than ripe ‘Arbequina’ fruits (slow-ripening cultivar), suggesting a prominent role of GAs during this process.

Similarly, the CK (*t*Z and iP) contents in olive fruit of both cultivars were high at stage 1 and gradually lowered thereafter, to the stage 5, except for *t*Z levels in ‘Arbequina’ fruit, which rose by 2.5-fold from stage 4 to 5 (Figure 3D), and for iP levels in ‘Picual’ fruit, which rose by 4.4-fold from stage 4 to 5 (Figure 3E). The levels of *t*Z were higher in the ripening ‘Arbequina’ fruit than in the ripening ‘Picual’ fruit, whereas the levels of iP were lower in the ripening ‘Arbequina’ fruit than in the ripening ‘Picual’ fruit. More specifically, this study indicates that the ripe ‘Arbequina’ fruit contained not only higher levels of IAA, but also high levels of CKs (*t*Z), suggesting that the high levels of endogenous auxins and CKs in ripe ‘Arbequina’ fruits are involved in a slow fruit ripening process and hence, a prolongated duration of the ripening process. Furthermore, the ABA levels increased during olive fruit ripening in both cultivars, but ABA levels of young ‘Arbequina’ fruits (stage 1) were low, but higher in young ‘Picual’ fruits (Figure 3F).

In the case of JA, in ‘Arbequina’ fruit, JA levels fell by 77% from stage 1 to 2 and remained unaltered afterwards, while JA levels rose 2.5-fold from stage 3 to 4 and fell by 63% from stage 4 to 5. Comparable to the trend during ‘Arbequina’ fruit growth, JA levels fell by 91% between stages 1 and 2 in ‘Picual’ fruit, but then rose 7.5-fold from stage 2 to 4, to descend by 63% afterwards (Figure 3G). However, SA levels rose during olive fruit growth, and then they fell at the maturation stage (stage 3) or the onset of ripening (stage 4), to remain constant afterwards in both olive cultivars (Figure 3H). Thus, these results show that both olive cultivars presented different accumulation dynamics of plant hormones during olive fruit development, suggesting that hormonal regulatory networks differ between olive cultivars and contribute to differences in the olive fruit size and shape as well as the ripening duration.

### 2.3. Transcriptional Regulation of Hormone-Related Genes during Olive Fruit Growth and Ripening

**Figure 4:**
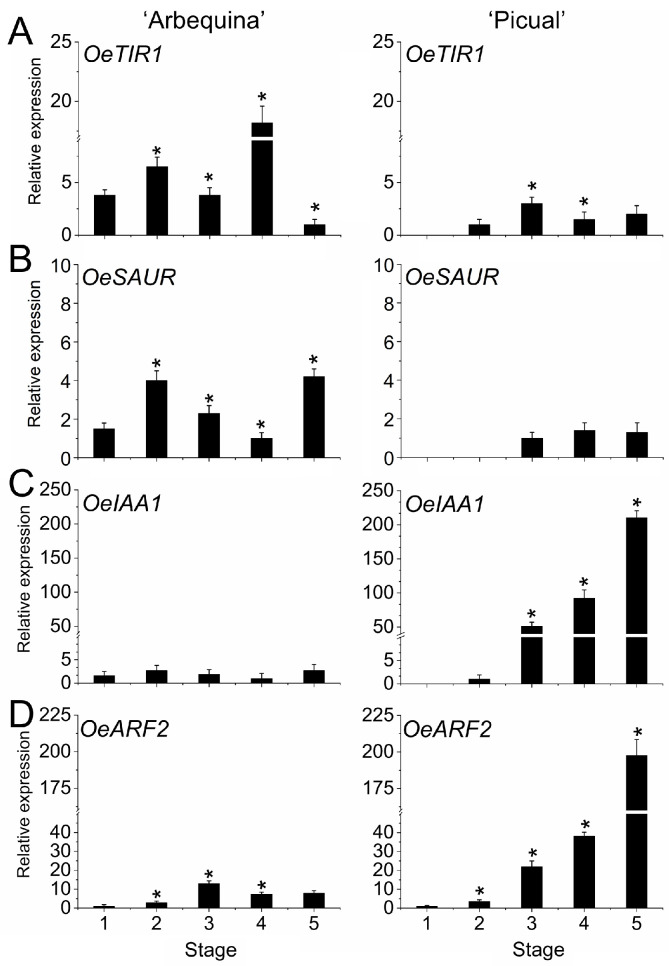
Expression of different components of the auxin signalling pathways during olive fruit growth and ripening. Expression patterns of *OeTIR1* (**A**), *OeSAUR* (**B**), *OeIAA1* (**C**), and *OeARF2* (**D**) during olive fruit development and ripening in ‘Arbequina’ and ‘Picual’ cultivars. Data are the means ± SD of three biological replicates with three technical repeats each and were found by qRT-PCR normalized against *Olea europaea* ubiquitin. Statistically significant differences from the preceding point based on Tukey’s test (*p* < 0.05) are denoted by asterisks.

As a means of gaining information concerning hormone dynamics during olive fruit development, an investigation was made into the possibility that hormone biosynthesis and signalling genes were differentially regulated during fruit growth and ripening in the two olive cultivars. The results showed that the expression of auxin signalling genes (*OeTIR1*, *OeIAA1*, *OeARF2*, and *OeSAUR*) followed a different pattern in ‘Arbequina’ with respect to ‘Picual’ fruits. That is, *IAA1* and *ARF2* genes were exclusively upregulated in ‘Picual’ olive fruit during fruit ripening but presented lower levels of expression in ‘Arbequina’ fruit (Figure 4).

In accordance with the IAA content, *OeTIR1* and *OeSAUR* genes showed greater expression at the stages 4 and 5, respectively, during fruit ripening in ‘Arbequina’ fruit (Figure 4A,B), whereas this did not occur in ‘Picual’ fruit. Thus, the IAA accumulation during olive fruit ripening clearly corresponded to the upregulation of *OeTIR1* and *OeSAUR* expression in ‘Arbequina’. These data therefore support the conjecture that the two olive cultivars may differ in the auxin signalling regulation during olive fruit ripening.

**Figure 5:**
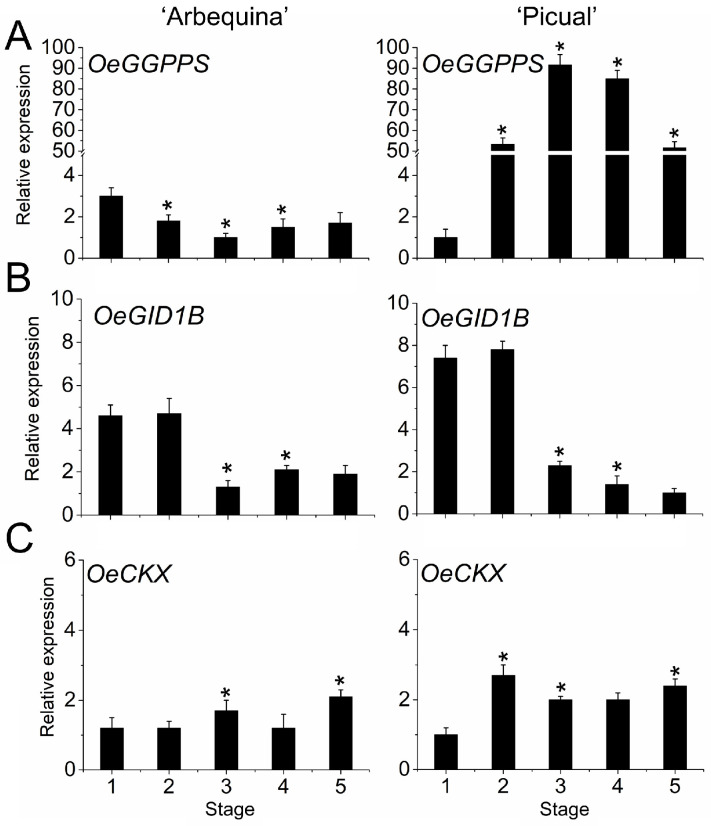
Expression of *OeGGPPS* (**A**), *OeGID1B* (**B**), and *OeCKX* (**C**) during olive fruit growth and ripening in ‘Arbequina’ and ‘Picual’ cultivars. Data are the means ± SD of three biological replicates with three technical repeats each and were gained by qRT-PCR normalized against *Olea europaea* ubiquitin. Statistically significant differences from the preceding point based on Tukey’s test (*p* < 0.05) are denoted by asterisks.

The data for GAs suggest that GA synthesis was upregulated by *OeGGPS*, coding geranylgeranyl pyrophosphate synthase (GGPS), during olive fruit ripening in ‘Picual’ fruit (Figure 5A). Meanwhile, we showed that GA signalling, regulated by *OeGID1B* receptors, was downregulated during olive fruit ripening in both cultivars (Figure 5B). Also, *CK oxidase/dehydrogenase* (OeCKX) gene expression increased in the fully ripe fruit of both cultivars, implying that the deactivation of CK may actively occur in ripe olive fruit (Figure 5C). In addition, *OeCKX* was upregulated at stages 2 and 3, in ‘Picual’ and ‘Arbequina’ fruits, respectively, parallel to the total CK content decrease.

**Figure 6:**
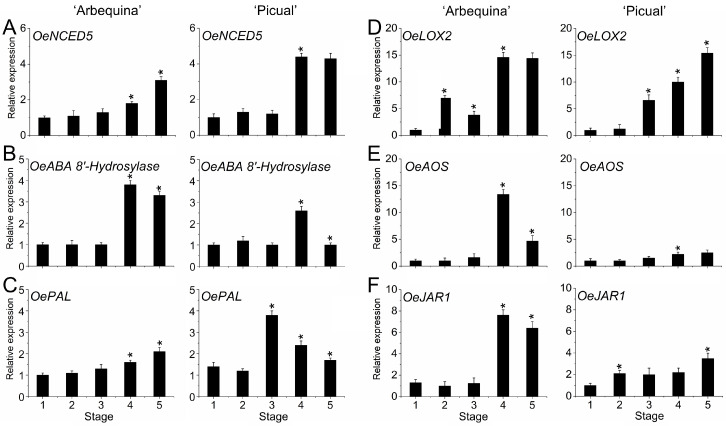
Expression of *OeNCED5* (**A**), *OeABA 8′-OH* (**B**), *OePAL* (**C**), *OeLOX2* (**D**), *OeAOS* (**E**), and *OeJAR1* (**F**) during olive fruit growth and ripening in ‘Arbequina’ and ‘Picual’ cultivars. Data are the means ± SD of three biological replicates with three technical repeats each and were obtained by qRT-PCR normalized against *Olea europaea* ubiquitin. Statistically significant differences from the preceding point based on Tukey’s test (*p* < 0.05) are denoted by asterisks.

Meanwhile, ABA contents increased during olive fruit ripening in both cultivars, and the levels of expression of gene coding for 9-cis-epoxycarotenoid dioxygenase (NCED), involved in ABA biosynthesis, were higher during olive fruit ripening in both olive cultivars (Figure 6A). Other transcripts involved in ABA catabolism, such as *ABA 8-OH*, which encodes ABA 8′-hydroxylase, was upregulated in both olive fruits at stage 4 (Figure 6B), in contrast to the ABA levels detected in olive fruit at this time.

In the case of SA, the expression of *PAL1*, which is associated with SA biosynthesis, was upregulated during fruit ripening in ‘Arbequina’ fruit (Figure 6C), while the SA level remained almost constant. Similarly, *PAL1* was upregulated only at the maturation stage in ‘Picual’ fruit, parallel to the decrease in SA content.

In addition, the expression of two genes coding for lipoxygenase (LOX) and allene oxide synthase (AOS), involved in JA synthesis, showed a pronounced increase at stage 4 in both olive cultivars (Figure 6D,E), in accordance with the surges in JA content observed in both cultivars at this stage, although these increases were greater in the case of the ‘Arbequina’ fruit. However, another gene encoding JAR1 (JASMONOYL ISOLEUCINE CONJUGATE SYNTHASE1), involved in JA-Ile biosynthesis, was upregulated at stage 4 followed by a decrease in the expression levels up to full fruit ripening (stage 5) in ‘Arbequina’ fruit, while *OeJAR1* increased in ‘Picual’ fruit at stage 5 (Figure 6F).

Therefore, distinct accumulation patterns of hormones and the expression of hormone-related genes in olive cultivars provided insights into the complex processes involved in olive fruit ripening, suggesting that distinct hormonal metabolism and signalling occur in this species during fruit ripening.

## 3. Discussion

**Figure 7:**
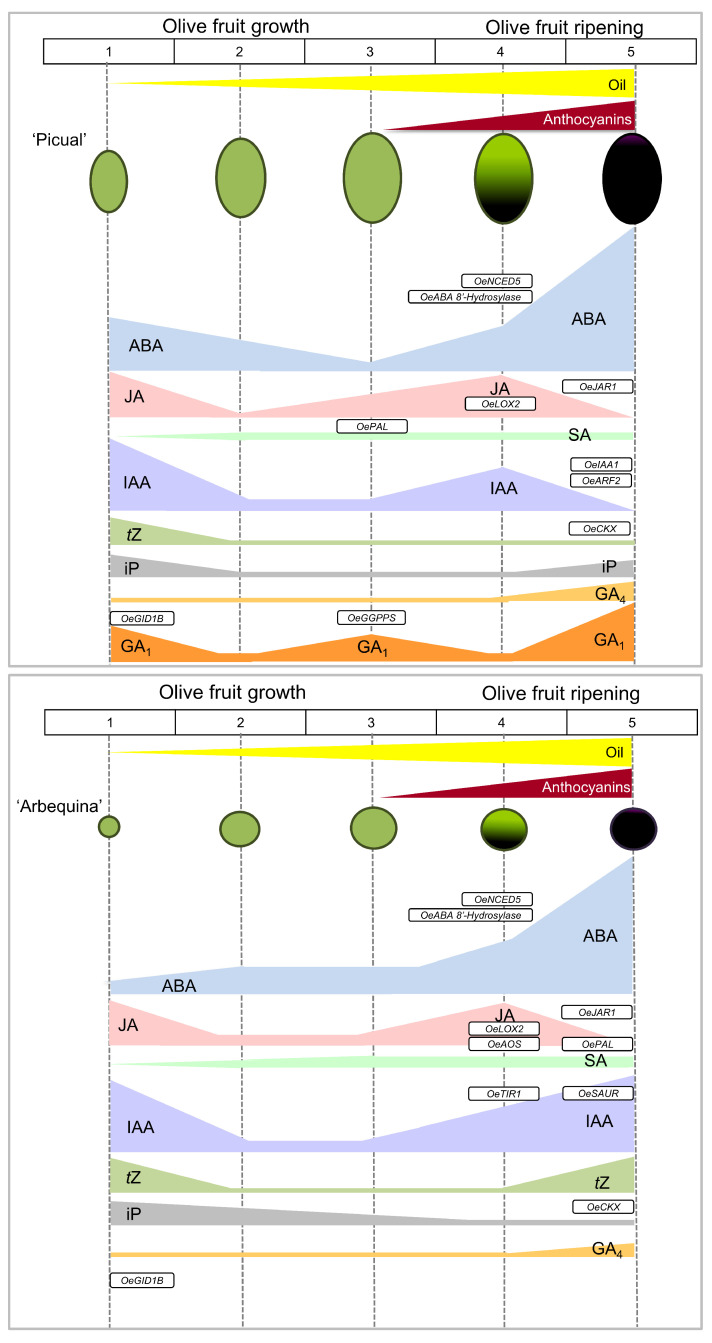
Scheme representing the hormone profiling of ‘Picual’ and ‘Arbequina’ olive cultivars during the stages of fruit development. See text for details.

Although the application of molecular and genomic approaches has been focused on the fruit development process in olives [49,50,55,62,64,65] for the enhancement of oil yield, information concerning the hormonal changes during fruit development is still limited for olives, in contrast to other non-climacteric fruits. So far, no studies have reported hormonal changes during fruit ripening in olives. Moreover, a key unresolved question regarding the olive fruit is whether olive cultivars share a common ripening mechanism and, if so, which of the known plant hormone(s) are involved. In the present study, the physiological and hormonal changes were examined in developing olive fruits of two major olive cultivars in commercial production for the oil market, ‘Picual’ and ‘Arbequina’, which differ in fruit size and shape, as well as fruit ripening rate and duration. In addition, both olive cultivars display significant phenotypic variation in ripe fruit abscission [77,78,79,80]. The results of the present study show that both olive cultivars presented different accumulation dynamics of plant hormones as well as different expression of some genes related to hormonal metabolism and signalling during key points of the stages of olive fruit development (Figure 7).

Here, the evaluation performed throughout the different development stages of olive fruit indicated a decrease in IAA, CK, and JA, and an increase in SA during fruit pulp growth in both olive cultivars. This coincided with the major increase in mesocarp cell expansion of olive fruit, i.e., 60% of final size of the mesocarp cell, as well as in fruit weight, resulting in great variation between the final size of the fruit in the two cultivars. In addition, these growth parameters agreed with the lipid accumulation in both cultivars. By contrast, throughout this pulp growth phase, the data indicate that the two olive cultivars differed in accumulation dynamics of GA_S_, suggesting underlying differences in fruit size and shape. Specifically, GA_1_ was undetectable in ‘Arbequina’ fruit (small, round fruit), whereas the GA_1_ level increased together with fruit size and weight in the ‘Picual’ cultivar (large, elongated fruit) during this phase. In addition, no significant changes of GA_4_ levels were found during fruit growth in either olive cultivar. Thus, unlike in small sized fruit, GA_1_ levels rose in the large sized fruit during growth by cell expansion after endocarp lignification, indicating that the presence of GA_1_ in ‘Picual’ fruit could be correlated to the fruit size of this cultivar in comparison to the ‘Arbequina’ cultivar. These results agree with the established notion that GA participates mainly in the cell expansion process in olive fruit [20]. In the ‘Picual’ cultivar, the level of GA_1_ was high at flowering and then fell during the cell division phase, while the levels of GA_1_ and GA_4_ peaked during the cell expansion phase of early ‘Picual’ fruit development, in agreement with findings elsewhere for the upregulated expression patterns of *OeGA3ox*, coding GA 3-oxidase (GA3ox), and *OeGID2*, coding GA receptor [20]. Likewise, in plums, during the fruit growth phase, when cell expansion is greatest, expression of three DELLAs is low, consistent with involvement of GA [81]. However, the mechanisms of GA action during olive fruit growth, after the endocarp lignification stage and before the onset of fruit ripening, remain to be elucidated. In the present study, the expression of GA receptor gene *OeGID1B* did not notably change in olive fruit between cultivars, whereas the expression of the GA biosynthesis gene *OeGGPS*, coding geranylgeranyl pyrophosphate synthase (GGPS) in olive fruit, increased consistently with the augment in GA_1_ content at stage 3 during fruit pulp growth in ‘Picual’ fruit. By contrast, *OeGGPS* expression was found to be repressed in the ‘Arbequina’ fruit from stages 1 to 3. Likewise, a significant difference was found between olive cultivars; the *OeGGPS* expression level was higher in ‘Picual’ than in ‘Arbequina’, indicating a relationship between the expression of *OeGGPS*, the cell expansion, and the subsequent increase in olive fruit size. In line with this, reported comparisons of transcriptomic analyses between varieties of small and large grape berries have shown that differentially expressed transcripts were related mainly to auxin, ABA, ethylene, and brassinosteroids (BRs), as well as to GAs [82]. Thus, the high expression of *OeGGPS* in ‘Picual’ might be correlated with the larger fruit size observed in this cultivar when compared to ‘Arbequina’, in accord with the GA accumulation profiles.

In other olive cultivars, several genes with annotated functions in GA biosynthesis and responses have been identified in the fruit transcriptome [53,55,66] but no available studies have investigated the hormonal contents of olive fruits during the same fruit developmental phase. Based on the data compiled, the present work poses the question as to whether this fruit growth mechanism of ‘Picual’ could be regarded as similar to that of other olive cultivars with large, elongated fruit or whether this specific fruit pulp growth regulation is unique to the cultivar ‘Picual’. Previous studies have shown that the manipulation of GA levels can influence both fruit size and morphology [49,52,83,84,85,86]. Moreover, a recent work has revealed tight connections between fruit shape variation and microtubules through integration of phytohormones, including GAs, auxin, and BRs [87]. Although many questions remain unanswered regarding the physiological basis that controls olive fruit size or shape, the present study shows that the level of endogenous GA_1_ and its biosynthesis may play a key role in the regulation of olive fruit growth (mainly by cell expansion) and eventually of olive fruit size and shape. However, further studies are needed to determine the molecular and genomic control underlying the regulation of GA pathways and interactions with other pathways during olive fruit growth in different olive cultivars.

In both olive cultivars, the fruit ripening process starts with a rise of the ABA level in parallel with the accumulation of anthocyanins and the decline of fruit firmness, then the expression of ABA-related genes, such as *OeNCED5* (9-cis-epoxycarotenoid dioxygenase) and *OeABA-8OH* or *OeClCYP707As* (abscisic acid 8′-hydroxylase) also increased, suggesting that both ABA biosynthesis and inactivation were stimulated during olive fruit ripening. Generally, ABA plays a pivotal role in non-climacteric fruit ripening [40,41,42,45], but this regulatory role of ABA during fruit ripening has been demonstrated in both non-climacteric and climacteric fruits [44,45,88]. Our results indicate that *OeNCED5* and *OeABA-8OH* are associated with ABA levels and olive fruit ripening. By contrast, in strawberry, ABA repressed the expression of *FveCYP707A4*a but promoted that of *FveNCED* during fruit ripening [85,89]. The ABA catabolism gene *FveCYP707A4a* was reported to be an important crosstalk point for auxin, GA, and ABA, regulating the transition from the early growth phase to the ripening phase [85]. In ripening olive fruit, the upregulation of NCED is presumably sufficient to maintain ABA at levels higher than those that stimulated fruit ripening in olives. In fact, in both fully ripe fruits, the most abundant hormone was ABA, but the ABA levels were higher in ‘Picual’ (fast-ripening) than in ‘Arbequina’ (slow-ripening). Notably, levels of *OeNCED5* transcripts were more than two-fold higher in ‘Picual’ olive fruit than in ‘Arbequina’ olive fruit at the onset of fruit ripening. During peach fruit ripening, the role of *NCED* genes, including *NCED5*, were observed in cooperatively regulating the ABA response [90]. Similarly, in the ‘Leccino’ olive cultivar, the synthesis of ABA was also stimulated throughout fruit ripening since a key enzyme that encoded AAO3 (ABSCISIC ALDEHYDE OXIDASE 3) was upregulated at the onset of fruit ripening [55]. The observed upregulation of genes involved in ABA biosynthesis is consistent with a higher ABA content amply reported in other non-climacteric fruits during fruit ripening [40,41,42,44,47,88,91,92]. Thus, it is proposed here that a considerable rise in the ABA level during olive fruit ripening might derive mainly from the increase in *OeNCED5* gene expression in both olive cultivars.

In olives, mainly anthocyanins are responsible for the black colour of ripe fruit, this ripening stage also being characterized by a significant increase in oil content [14]. Here, both contents had similar behaviour, actively operating during olive fruit ripening. The final changes in fruit colouring are a key stage during the olive fruit ripening, after which oil accumulation processes cease [93]. In previous studies, the exogenous application of ABA has been reported to raise anthocyanin levels and promote fruit ripening [88,94,95]. However, exogenous ABA application did not show a promotion of fruit ripening in the two olive cultivars when ABA was included in the pre-harvest treatment at the onset of fruit ripening to produce olive fruits enriched in antioxidants [73]. By contrast, exogenous applications of methyl jasmonate (MeJA) on the olive tree have been associated with anthocyanin accumulation, supporting the contention that MeJA has a role in inducing ripening in olives [74], as previously demonstrated in other non-climacteric fruits [96,97,98].

JA and JA-Ile contents may be crucial in the beginning of fruit ripening, and their contents begin to decline at the late ripening stage in non-climacteric fruits [91,99]. In the present study, a similar trend for JA level is reported, exhibiting higher concentrations of endogenous JA at the onset of olive fruit ripening followed by a decline during fruit ripening in both olive cultivars. Moreover, genes involved in the JA metabolism (*OeJAR1*) and biosynthesis (*OeLOX2* and *OeAOS*) were upregulated at the onset of ripening, consistent with JA levels in olive fruits of both olive cultivars. Indeed, previous work has shown that genes involved in JA metabolism were also upregulated at the onset of fruit ripening in the olive cultivar ‘Leccino’ [55]. Although the possible role of JA in olive fruit ripening remains largely uncharacterized, the increase in JA and gene expression levels coincided with the onset of olive fruit ripening in both of the olive cultivars used in the present study, suggesting that JA is involved in olive fruit ripening. Previously, an antagonistic relationship between the JA and the ABA pathways during non-climacteric strawberry fruit ripening has been proposed [99]. However, it has been shown that endogenous MeJA might stimulate ABA levels in grape berries, due to MeJA activated lipoxygenase, which is involved in ABA synthesis [100], while a similar effect of MeJA on ethylene synthesis in climacteric fruits has been reported [101,102]. The experimental data of the present study support the hypothesis that JA plays a role in the onset of olive fruit ripening through stimulation of ABA biosynthesis at the transcriptional level, but it is possible that JA acts antagonistically towards ABA during olive fruit ripening, as shown in other non-climacteric fruits [99].

It has been shown that, during fruit ripening, the metabolism of ABA, JA, and IAA differ between grape cultivars [48]. In the present study, the results indicate that the start of the rise in ABA levels just at the onset of olive fruit ripening coincides with the higher JA and IAA levels in both olive cultivars, but an inverse relationship between IAA and GA levels was apparent between cultivars during olive fruit ripening. The IAA level continued to rise in ‘Arbequina’ fruit during slow-ripening (from stage 4 to 5) along with the CK (*t*Z) accumulation, whereas the IAA level decreased concomitantly with an increase in GAs (GA_1_ and GA_4_) in ‘Picual’ fruit during fast-ripening (stages 4–5). Thus, IAA and GAs presented different accumulation patterns during olive fruit ripening. The fact that the slow-ripening olive cultivar accumulated IAA and CK from the onset of fruit ripening until full ripening in contrast to the fast-ripening olive cultivar, which undergoes GA accumulation, suggests that IAA and CK retard the ripening process in the slow-ripening olive cultivar. Generally, the promotion of fruit growth by auxin and CK, and their inhibition of fruit ripening, are highly conserved in plant species [40,43], but recent evidence indicates that auxin and CK may play a complex role in regulating ripening through interactions with other plant hormones, supporting the contention of the activation of auxin and CK signalling during this stage [44,103]. In apples, it has been shown that the genotype-specific feature of auxin metabolism in maturing fruit may determine the different rates of ripening progression [104]. In this regard, the results of the IAA levels in ‘Picual’ fruit were similar to those reported in tomatoes, climacteric fruits, and in strawberries, non-climacteric fruits, where the IAA level increased at the onset of fruit ripening and then decreased during the progression of fruit ripening [40,43]. However, in ‘Arbequina’, no rising or falling pattern was seen in the two studies prior to the present one, revealing that IAA content is high when the ripe fruits are most slowly ripening in olives. It appears that the IAA involves counteracting the ABA effect, thereby circumventing excessive ABA signal amplification. In grapes, an ABA-IAA switch reportedly controls the degree to which a bunch of grapes ripens [105], and the level of both hormones augment simultaneously in sweet cherries [51]. Moreover, genes related to auxin signalling exhibited varying expression patterns between olive cultivars, showing an increased fruit *OeTIR1* and *OeSAUR* expression associated with slow-ripening and with high IAA level, while the expression of the *OeIAA1* and *OeARF2* genes increased markedly during fast-ripening. In tomatoes, altered *ARF2* expression leads to a change in ABA, CK, and SA contents highlighting that auxin signalling intersects hormonal signals in the regulation of fruit ripening [103]. Therefore, differential regulation of auxin pathway genes regulates olive fruit ripening in fast- and slow-ripening cultivars, suggesting that auxin signalling involves different regulatory mechanisms depending on the olive cultivar.

Previous studies have shown GAs to be associated with fruit ripening [83,86,106,107,108]. In non-climacteric fruit, GA and ABA often counteract each other in the regulation of the fruit ripening [85]. In the present study, ‘Picual’ fruit registered the highest GA_1_ and GA_4_ levels at the fully ripe stage and rose around 11- and 22-fold, respectively, from the onset of fast-ripening. In contrast, the relative lack of GA_1_ and/or reduced GA-sensitivity in ‘Arbequina’ appeared to be at least partially responsible for prolonged fruit ripening in this cultivar. Thus, while IAA levels exhibited a decline during fruit fast-ripening, the opposite pattern was detected for GA (GA_1_ and GA_4_), suggesting that GA plays a key role in progressive olive fruit ripening, since the presence of endogenous GA in olive fruit shortened the ripening duration. Similarly, high levels of GA_1_ were detected throughout fruit ripening in certain stone fruits [106,107]. However, despite a substantial increase in the endogenous GA_1_ and GA_4_ levels exclusively during fruit ripening in ‘Picual’ fruit, *OeGGPS* and *OeGID1B* expression levels did not rise, emphasizing a complex regulation of the GA metabolism and signalling in developing olive fruits. In addition, in ‘Arbequina’ fruit (slow ripening), IAA and CK (*t*Z) accumulated during olive fruit ripening, suggesting that GA accumulation could coordinate ripening duration by lowering auxin and CK (*t*Z) levels. In particular, GA (GA_3_) and CK (Z) contents fluctuated and differed between two strawberry cultivars during fruit ripening [50], highlighting the value of characterizing multiple genotype-specific fruit ripening patterns in a species. The present work shows that olive fruit ripening can have opposing effects on the levels of GAs (GA_1_ + GA_4_) and of IAA + CK (*t*Z) for a specific olive cultivar, revealing that the genotype-specific feature of the GA_1_ presence in ripening olive fruit may determine the different rate of fruit ripening progression. Consequently, the present study indicates that GA_1_ could play a dual regulatory role in olives, firstly promoting the growth of olive elongated fruit mainly by cell expansion, and secondly, participating afterwards in shortening the fruit ripening duration. These results agree with findings elsewhere [86] indicating that the manipulation of GA levels can simultaneously influence tomato fruit shape and ripening, implying that a common regulatory mechanism exists in different plant species. Furthermore, GA-deficient mutant fruits present with several developmental disorders, including reduced fruit size, and delayed ripening [109,110,111]. Although the role of GA in the regulation of olive fruit development deserves more attention in the future, the present findings lead to the hypothesis that the differences in the fruit morphology and the ripening duration observed in the two olive cultivars are, at least in part, due to variations in the GA content and/or responsiveness in olive fruit. Overall, the present findings reveal that ripening occurs in association with a rise in IAA and *t*Z levels together with an increase in ABA in slow-ripening fruits, whereas GA and ABA act synergically towards fast-ripening of olive fruits. Therefore, in addition to ABA, the results of the present study indicate that IAA, CK, and GA are hormones involved in olive fruit ripening, and their regulatory roles differ between fast- and slow-ripening fruits in olives.

## 4. Materials and Methods

### 4.1. Plant Material and Cytological Analysis

Olive trees (*Olea. europaea* L. cv. ‘Picual’ and ‘Arbequina’) grown under drip irrigation and fertirrigation during the 2018 and 2019 growing seasons in an orchard near Badajoz (Spain) were studied. Fruits at five specific developmental stages (‘Picual’: 63, 100, 150, 171 and 200 DPA; ‘Arbequina’: 63, 100, 150, 164 and 240 DPA) were randomly tagged, collected, and processed as previously described [61]. The two cultivars studied, ‘Arbequina’ and ‘Picual’, with different final fruit sizes and shapes [5,25], required different days after green fruit maturation to reach their full fruit ripening (90 and 50 days, respectively). A total of 250 fruits from 10 olive trees per cultivar were used for each stage. As a means of minimizing the effects related to asynchronous fruit ripening within the same tree, fruits with similar pigmentation were picked from all around the external parts of the tree canopy. Whole fruits of ‘Arbequina’ and ‘Picual’ olive cultivars were weighed, and the longitudinal and equatorial diameters were measured at different stages. Fruit firmness was measured at four equatorial regions of the fruit using a penetrometer (model SMT-T-50, Toyo Baldwin, Tokyo, Japan) fitted with a 5 mm plunger [61]. An initial group of pericarp samples from different stages was used for cytological analysis, while another group was immediately frozen in liquid nitrogen and stored at −80 °C for the determination of total lipids, anthocyanins, and hormones, as well as for RNA extractions.

The cytological study was performed as described elsewhere [72]. For this, fruits (3 fruits/stage) were cut at the equatorial plane. Sections (3 mm) cut with glass knives were stained with 0.04% (*w*/*v*) toluidine blue and photographed using a Zeiss Axiophot microscope (Zeiss, Oberkochen, Germany) coupled to a Spot digital camera (Diagnostic Instruments Inc., Sterling Heights, MI, USA).

### 4.2. Extraction of Total Lipids and Anthocyanins

Total lipids were extracted from 800 mg fruit pericarps of freeze-dried powder following a protocol described elsewhere [61]. Extracted lipids for each stage of development from three different extractions were quantified gravimetrically after evaporation until dry, under nitrogen at 40 °C.

Total anthocyanins were extracted from fruit pericarps and determined following the method reported elsewhere [112]. All experiments were conducted with at least three biological replicates. Sodium metabisulphite (0.2 g) was added to olive paste (10 g) prior to homogenization. The olive paste was then mixed with 15 mL of methanol:water (80:20) in a Ultraturrax for 2 min. The resulting slurry was centrifuged at 9000× *g* for 10 min, after which the supernatant was collected and poured into a 50 mL volumetric flask. From the residue, the extraction with 15 mL of methanol:water was repeated twice. The resulting supernatants were added to the first one and the flask was made up to volume with methanol:water. For the spectrophotometrical determination of the total anthocyanins, 25 mL of the sample extract was evaporated to dryness in Rotavapor and the residue was dissolved in 5 mL of a solution of potassium chloride-hydrochloric acid (pH 2). The procedure was as follows: 1 mL of the extracted sample was placed in a beaker, to which 1 mL of 0.1% hydrochloric acid in 95% ethanol and 20 mL of an aqueous solution of 2% hydrochloric acid were added; 10 mL of this solution was transferred into two glass tubes and 4 mL of a solution of 15% sodium metabisulphite was added to the first and 4 mL of water to the second. An absorbance reading was made at 520 after 20 min for each glass tube and the difference between the two readings was calculated.

### 4.3. Quantification of Plant Hormones

The plant hormones were quantified as described elsewhere [20]. A pool of 100 mg fresh weight/sample was used for each measurement, split into three independent biological replicates per sample. Aliquots of lyophilized material were extracted with 80% methanol-1% acetic acid. Deuterium-labelled hormones (purchased from Prof. L Mander-Canberra, OlChemim Ltd-Olomouc, or Cambridge Isotope Lab, Andover, MA, USA) [17,17-^2^H]GAn, [^2^H_5_]IAA, and [^2^H_6_]ABA were added as internal standards for the quantification of SA and ABA. For the quantification of JA, the compound dhJA was used instead. For collecting the acid fractions containing SA, ABA, and JA, the extracts were passed consecutively through HLB (reverse phase), MCX (cationic exchange), and WAX (ionic exchange) columns (Oasis 30 mg, Waters, MA, USA) were used. The final residue was dissolved in 5% acetonitrile-1% acetic acid, and the hormones were separated by reverse phase UPHL chromatography (2.6 μm Accucore RP-MS column, 100 mm length × 2.1 mm i.d., ThermoFisher Scientific, Waltham, MA, USA) with a 5% to 50% acetonitrile gradient. The hormones were analysed by electrospray ionization and targeted-SIM using a Q-Exactive spectrometer (Orbitrap detector, ThermoFisher Scientific, Waltham, MA, USA). The concentrations of hormones in the extracts were determined with the use of embedded calibration curves and the Xcalibur 4.1 SP1 build 48 and TraceFinder programs.

### 4.4. RNA Extraction and Quantitative Real-Time PCR Analysis

Total RNA extraction and qRT-PCR assays from fruit samples were performed as indicated elsewhere [65]. A total of 13 olive genes encoded for key proteins involved in hormonal metabolism and signalling (auxin, GA, CK, ABA, SA, and JA) were previously identified, on the basis of homology, by RNA-Seq analysis in ‘Picual’ fruit [65] and tested for expression analysis in ‘Picual’ and ‘Arbequina’ olive fruit by qRT-PCR in the present study. The qRT-PCR assays were performed with gene-specific primers (Table S1). The cDNA was amplified using a SYBRGreenPCR Master kit (Applied Biosystems, Madrid, Spain) containing an AmpliTaq Gold polymerase on an iCycler (BioRad, Munich, Germany), following the protocol provided by the supplier. Samples were subjected to thermal cycling conditions of DNA polymerase activation at 94 °C, 45 s at 55 °C, 45 s at 72 °C, and 45 s at 80 °C; a final elongation step of 7 min at 72 °C was performed. The melting curve was designed to increase by 0.5 °C every 10 s from 62 °C. The amplicon was analysed by electrophoresis and sequenced once for confirmation of identity. A calibration dilution curve and slope calculation were used to estimate qRT-PCR efficiency. Expression levels were determined as the number of cycles needed for the amplification to reach a threshold fixed in the exponential phase of the PCR (CT). The data were normalized for the quantity of the *O. europaea* ubiquitin (*OeUB*) gene [5]. In two independent experiments, duplicates were used from three biological replicates.

### 4.5. Statistical Analysis

All the experiments were performed in triplicate, and all data are presented as mean ± standard deviation. Variables of three replicates were compared using Tukey’s multiple-range test, and a *p* value at 0.05 was considered significant.

## 5. Conclusions

This detailed study was undertaken to provide a comprehensive description of the hormonal profiling during olive fruit growth and ripening. Olive fruit ripening depends mainly on ABA, but the IAA, CK, and GA contents and/or responsiveness differ between olive cultivars during fruit ripening. This is the first report available describing the regulation of hormonal content and gene expression during olive fruit ripening. Further studies are in progress to assess the relevance of these hormones in other olive cultivars. In particular, the differences in GA content in the olive cultivars studied cannot be explained by the differences in expression of the protein GGPPS, this presumably results from more complex metabolic regulation involving biosynthesis, catabolism, and conjugation. The relative lack of GA_1_ and/or reduced GA sensitivity in the ‘Arbequina’ cultivar appears to be at least partially responsible for the smaller fruit size and prolonged fruit ripening duration in this cultivar, suggesting that the fruit size and ripeness of olives can be regulated by regulating GA. However, it cannot be ruled out that other hormonal or physiological signals, not yet elucidated, can influence some aspects of the olive fruit growth and ripening process.

## Data Availability

The data presented in this study are available in the Supplementary Materials.

## Supplementary Materials

The following supporting information can be downloaded at: https://www.mdpi.com/article/10.3390/plants12223832/s1, Table S1: PCR-primers used in this study.
