# Supplementary material for: Hormonal Content and Gene Expression during Olive Fruit Growth and Ripening
Source: Plants (Basel). 2023 Nov 12;12(22):3832. doi: 10.3390/plants12223832 (PMC10675085; doi:10.3390/plants12223832)
Supplement: Supplementary file 1 [file plants-12-03832-s001.zip › plants-2655695-supplementary.pdf]

SUPPLEMENTARY DATA

Table S1. PCR-primers used in this study.

Primer	Sequence	Gene
GID1B-F	5′-AGATTGGTTCCACTTAATACCTGG-3′	*OeGID1B*
GID1B-R	5′-ATGAGTAAAGCTTCCCCCGTGGAA-3′	
GGPPS-F	5′-GAAGGACTAGTGGCTGGACAGGTA-3′	*OeGGPPS*
GGPPS-R	5′-AAACAAGAGCCCAATACACCTAGC-3′	
PAL-F	5′-GATATATTGAAGCTCATGTCGTCT-3′	*OePAL*
PAL-R	5′-AAGAACTTGCCTCAATTTTTGCAT-3′	
CKX-F	5′-AAATGGATCAGAGTGCTTTACTCA-3′	*OeCKX*
CKX-R	5′-TGGGTGTGGAAGATCCCACAATCC -3′	
JAR1-F	5′-GATTTGCAGCTGGCTGTAGAAGCA-3′	*OeJAR1*
JAR1-R	5′-TTTGAATTGGCTAACAGCAGCTCC-3′	
ABA-8OH-F	5′-ATGCCATTTGGCAGTGGAGTACAT-3′	*OeABA-8OH*
ABA-8OH-R	5′-GGTTGGTTCTTGTTTCCAAAATAT-3′	
AOS-F	5′-TCCCAACATGCTTAAATGGGTTGGT-3′	*OeAOS*
AOS-R	5′-GGATTCAATCACGAAGTCGCGTTT-3′	
LOX2-F	5′-GGCCATGCAGACAAGAAGGATGA-3′	*OeLOX2*
LOX2-R	5′-CCCGAGATACTCCTCGTCGGGCGA-3′	
NCED5-F	5′-AACCTAGAAGCCGGTATGGTTAAC-3′	*OeNCED5*
NCED5-R	5′-CCCGTGGAACCCGTATGGGACTCT-3′	

ARF2-F	5′-ATGCCGCCTGGTCATTCAAGAGAA-3′	*OeARF2*
ARF2-R	5′- CTTGGCAAGATCAACAGACCTGCC-3′	
SAUR-F	5′-CAAACAGCAGTTTTGAAGCAAATT-3′	*OeSAUR*
SAUR-R	5′-TCGAAGCATTGACGTTAGAGATCG-3′	
TIR1-F	5′-CCAATGGATGAAGGCTTTGGA-3′	*OeTIR1*
TIR1-R	5′-GTCACCAGCAAAAGCAACTGACA -3′	
IAA1-F	5′-GGATGGCCTCCAGTGAGATCATAC-3′	*OeIAA1*
IAA1-R	5′- AGGGACATCTCCAACTAACATCCA -3′	
